# 

**Published:** 1829-11

**Authors:** 


### MONTHLY LIST OF MEDICAL BOOKS.

[*Medical Works cannot be entered on this List except a copy be sent for the purpose; the titles of Books having frequently been transmitted to us, as published, which have not appeared for weeks, or even months, after.*]

An Estimate of the real Therapeutic Value of all the new Chemical and other Remedies introduced into Medicine within the last twenty years, derived from actual Practice; with Directions for preparing and employing those which are worthy the attention of the Medical Profession. By RICHARD REECE, M.D.

Anatomy of the Human Bones, Joints, and Ligaments; arranged in eight Tables. For the use of Students.—Burgess and Hill.

These tables, which are very moderate in price, are particularly well adapted as references for the student to refresh and strengthen his memory, after he has perused more elaborate anatomical works.

The Art of Prolonging Human Life; in which the subject is fully considered, both philosophically and practically. By C. W. HUFELAND, M.D. first Physician to the King of Prussia, &c. A new Edition; with Notes, by an English Physician.—8vo. pp. 328. Simpkin and Marshall, London, 1829.

The talent of Hufeland is too well known to require our formal praise. From the perusal of this long-celebrated work, the medical and general student will derive instruction and amusement. It has already passed through several editions, and the present is improved by many interesting notes from an intelligent, although anonymous, editor.

A Manual of General Anatomy; or, a concise Description of the primitive Tissues and Systems which compose the Organs in Man. By A. L. J. BAYLE, D.M.P. &c. and H. HOLLARD, D.M.P. &c. Translated from the French, by HENRY STORER.—12mo. pp. 318. Wilson, London, 1829.

In this country we abound with works of all classes and all degrees of merit on descriptive anatomy, but our press has been much less fertile in the production of treatises on "General Anatomy." Until the appearance of this "Manual," indeed, we had no work in so condensed a form upon this important branch of medical science. Mr. Storer has shown his judgment in the selection of an original, and has executed the duties of a translator with fidelity and ability. This little work will be consulted with much advantage by every anatomical student.

A New Method of treating Burns and Scalds. By MICHAEL WARD, M.D. S.R.C.C.L.—Manchester.

A Treatise on Neuralgic Diseases dependent upon Irritation of the Spinal Marrow and Ganglia of the Sympathetic Nerve. By THOMAS P. TEALE, Surgeon; Senior Surgeon to the Leeds Public Dispensary, &c.—8vo. pp. 120. Highley, London, 1829.

Elements of General Anatomy, containing an Outline of the Organization of the Human Body. By R. D. GRAINGER, Lecturer on Anatomy and Physiology.—8vo. pp. 526. Highley, London, 1829.

A Letter to Lord Robert Seymour, with a Report of the Number of Lunatics and Idiots in England and Wales. By Sir ANDREW HALLIDAY, K.H. and M.D.—Underwood, 1829.

An interesting document to those engaged in making statistic researches into the number and condition of the insane in England and Wales.

Notions of the Nature of Fever and of Nervous Action. By W. FORRESTER BOW, M.D. &c.—8vo. pp. 100. Longman, London, 1829.

### NOTICES.

*Communications have been received from* Mr. BURNETT *and* Mr. LAWTON.

*If* "A Friend to Humanity" *will forward his name to the Editors, and consent to its being attached to his communication, they will publish it in the next Number. It is true the paper referred to is* professedly *a practical discussion, but it is also very obviously a covert attack upon the conduct of an individual, as the medical officer of a public institution; and therefore cannot be admitted anonymously.*

"CHIRURGUS" *requests us to inform him whether he can "safely apply the term original to his speculation." We answer, he cannot.*

